# Supplementary material for: In Silico Logical Modelling to Uncover Cooperative Interactions in Cancer
Source: Int J Mol Sci. 2021 May 5;22(9):4897. doi: 10.3390/ijms22094897 (PMC8125147; doi:10.3390/ijms22094897)
Supplement: Supplementary file 1 [file ijms-22-04897-s001.zip › ijms-1144228-supplementary.pdf]

Supplementary Tables

Unperturbed model, RPTP_L=1

RPTP_L	HGF	EGF	ECM	TGFB	IL6	DELTA	ROS	WNT	Existing phenotypes	Reachability probability	#Successful runs	#Runs
1	0	0	0	0	0	0	0	0	20	1.0000	N/A	N/A
1	1	0	0	0	0	0	0	0	20	1.0000	N/A	N/A
1	0	1	0	0	0	0	0	0	20	1.0000	N/A	N/A
1	0	0	1	0	0	0	0	0	20	0.6262	1.00E+05	1.00E+05
1	0	0	1	0	0	0	0	0	23	0.1805		
1	0	0	1	0	0	0	0	0	03	0.1933		
1	0	0	0	1	0	0	0	0	01	1.0000	N/A	N/A
1	0	0	0	0	1	0	0	0	21	0.4695	1.00E+05	1.00E+05
1	0	0	0	0	1	0	0	0	01	0.5305		
1	0	0	0	0	0	1	0	0	20	1.0000	N/A	N/A
1	0	0	0	0	0	0	1	0	02	1.0000	N/A	N/A
1	0	0	0	0	0	0	0	1	20	1.0000	N/A	N/A

NOTCH E1, RPTP_L=1

RPTP_L	HGF	EGF	ECM	TGFB	IL6	DELTA	ROS	WNT	Existing phenotypes	Reachability probability	#Successful runs	#Runs
1	0	0	0	0	0	0	0	0	20	1.0000	N/A	N/A
1	1	0	0	0	0	0	0	0	20	1.0000	N/A	N/A
1	0	1	0	0	0	0	0	0	20	1.0000	N/A	N/A
1	0	0	1	0	0	0	0	0	20	0.6210	99999	1.00E+05
1	0	0	1	0	0	0	0	0	03	0.3790		
1	0	0	0	1	0	0	0	0	01	1.0000	N/A	N/A
1	0	0	0	0	1	0	0	0	01	1.0000	N/A	N/A
											N/A	N/A
1	0	0	0	0	0	1	0	0	20	1.0000	N/A	N/A
1	0	0	0	0	0	0	1	0	02	1.0000	N/A	N/A
1	0	0	0	0	0	0	0	1	20	1.0000	N/A	N/A

Unperturbed model, RPTP_L=0

RPTP_L	HGF	EGF	ECM	TGFB	IL6	DELTA	ROS	WNT	Existing phenotypes	Reachability probability	#Successful runs	#Runs
0	0	0	0	0	0	0	0	0	20	1.0000	N/A	N/A
0	1	0	0	0	0	0	0	0	12	0.9370	1.00E+05	1.00E+05
0	1	0	0	0	0	0	0	0	02	0.0630		
0	0	1	0	0	0	0	0	0	12	0.8191	N/A	N/A
0	0	1	0	0	0	0	0	0	02	0.1809	N/A	N/A
0	0	0	1	0	0	0	0	0	20	0.6081	99997	1.00E+05
0	0	0	1	0	0	0	0	0	03	0.3919		
0	0	0	0	1	0	0	0	0	02	1.0000	N/A	N/A
0	0	0	0	0	1	0	0	0	21	0.4701	1.00E+05	1.00E+05
0	0	0	0	0	1	0	0	0	01	0.5299		
0	0	0	0	0	0	1	0	0	20	1.0000	N/A	N/A
0	0	0	0	0	0	0	1	0	02	1.0000	N/A	N/A
0	0	0	0	0	0	0	0	1	20	1.0000	N/A	N/A

NOTCH E1, RPTP_L=0

RPTP_L	HGF	EGF	ECM	TGFB	IL6	DELTA	ROS	WNT	Existing phenotypes	Reachability probability	#Successful runs	#Runs
0	0	0	0	0	0	0	0	0	20	1.0000	N/A	N/A
0	1	0	0	0	0	0	0	0	02	1.0000	N/A	N/A
											N/A	N/A
0	0	1	0	0	0	0	0	0	02	1.0000	N/A	N/A
											N/A	N/A
0	0	0	1	0	0	0	0	0	20	0.6017	99982	1.00E+05
0	0	0	1	0	0	0	0	0	03	0.3983		
0	0	0	0	1	0	0	0	0	02	1.0000	N/A	N/A
0	0	0	0	0	1	0	0	0	01	1.0000	N/A	N/A
											N/A	N/A
0	0	0	0	0	0	1	0	0	20	1.0000	N/A	N/A
0	0	0	0	0	0	0	1	0	02	1.0000	N/A	N/A
0	0	0	0	0	0	0	0	1	20	1.0000	N/A	N/A

Wild type, HGF=1 and ECM=1

RPTP_L	HGF	EGF	ECM	TGFB	IL6	DELTA	ROS	WNT	Existing phenotypes	Reachability probability	#Successful runs	#Runs
1	1	0	1	0	0	0	0	0	20	0.6226	1.00E+05	1.00E+05
1	1	0	1	0	0	0	0	0	23	0.1808		
1	1	0	1	0	0	0	0	0	03	0.1966		
0	1	0	1	0	0	0	0	0	03	1.0000	N/A	N/A

Wild type, EGF=1 and ECM=1

RPTP_L	HGF	EGF	ECM	TGFB	IL6	DELTA	ROS	WNT	Existing phenotypes	Reachability probability	#Successful runs	#Runs
1	0	1	1	0	0	0	0	0	20	0.6249	1.00E+05	1.00E+05
1	0	1	1	0	0	0	0	0	23	0.1798		
1	0	1	1	0	0	0	0	0	03	0.1953		
0	0	1	1	0	0	0	0	0	03	1.0000	N/A	N/A

Table S1: The EMT logical model predicts cooperation between RPTP_L, microenvironment signals and NOTCH E1. Probabilities of reaching EMT phenotypes (columns existing phenotypes), according to the model read-outs AJ and FA, starting from an E1 phenotype, when RPTP_L is fixed at 1 (upper left table), or RPTP_L is fixed at 0 (middle left panel), or HGF and ECM are fixed at 1 (lower left table), or RPTP-L is fixed at 1 in the presence of a NOTCH E1 mutation (upper right table), or RPTP-L is fixed at 0 in the presence of a NOTCH E1 mutation (middle right table), or EGF and ECM are fixed at 1 (lower right table). Green colors denote when the levels of the microenvironmental inputs is set to 1.

		UN		M1		M2		M3		10		11		H2		13		E1		H1		22		H3	
		Sgle	Dble	Sgle	Dble	Sgle	Dble	Sgle	Dble	Sgle	Dble	Sgle	Dble	Sgle	Dble	Sgle	Dble	Sgle	Dble	Sgle	Dble	Sgle	Dble	Sgle	Dble
	NOTCH E1		NA		NA		NA		NA		NA		NA		NA		NA		NA		NA		NA		NA
1	AKT KO																								
2	AKT E1																								
3	BCatAJ KO																								
4	BCatAJ E1																								
5	BCat KO																								
6	BCat E1																								
7	CK1 KO																								
8	CK1 E1																								
9	CSL KO																								
10	CSL E1																								
11	DVL KO																								
12	DVL E1																								
13	ECadAJ KO																								
14	ECadAJ E1																								
15	ECadAJ E2																								
16	ECad KO																								
17	ECad E1																								
18	EGFR KO																								
19	EGFR E1																								
20	ERK KO																								
21	ERK E1																								
22	FAK_SRC KO																								
23	FAK_SRC E1																								
24	FAK_SRC E2																								
25	FAT4 KO																								
26	FAT4 E1																								
27	GSK3B KO																								
28	GSK3B E1																								
29	HGFR KO																								
30	HGFR E1																								
31	HIF1a KO																								
32	HIF1a E1																								
33	ILK KO																								
34	ILK E1																								
35	ITG_AB KO																								
36	ITG_AB E1																								
37	JAK KO																								
38	JAK E1																								
39	JNK KO																								
40	JNK E1																								
41	LATS KO																								
42	LATS E1																								
43	MEK KO																								
44	MEK E1																								
45	miR200 KO																								
46	miR200 E1																								
47	NFkB KO																								
48	NFkB E1																								
49	p120AJ KO																								
50	p120AJ E1																								
51	PAK KO																								
52	PAK E1																								
53	PI3K KO																								
54	PI3K E1																								
55	RAF1 KO																								
56	RAF1 E1																								
57	RAP1 KO																								
58	RAP1 E1																								
59	RAS KO																								
60	RAS E1																								
61	RPTP KO																								
62	RPTP E1																								
63	SLUG KO																								
64	SLUG E1																								
65	SMAD KO																								
66	SMAD E1																								
67	SNAIL KO																								
68	SNAIL E1																								
69	STAT3 KO																								
70	STAT3 E1																								
71	TCF_LEF KO																								
72	TCF_LEF E1																								
73	TGFBR KO																								
74	TGFBR E1																								
75	YAP-TAZ KO																								
76	YAP-TAZ E1																								
77	ZEB KO																								
78	ZEB E1																								

Table S2: Phenotypes compatible with the 80 LoF and GoF single mutants and by the 78 combinations of double mutants involving a NOTCH GoF mutation. Each column corresponds to phenotypes compatible with each single LoF (KO) and GoF (E1) mutant indicated on the left, combined (Dble) or not (Sgle) with the NOTCH E1 mutation. Grey boxes indicate phenotypes retrieved by the model under the specified condition. For some perturbations, the phenotypic landscapes are reshaped, leading to the appearance of the novel unnamed phenotypes 10, 11, 13 and 22.
